# ATMIN is required for the ATM-mediated signaling and recruitment of 53BP1 to DNA damage sites upon replication stress

**DOI:** 10.1016/j.dnarep.2014.09.001

**Published:** 2014-12

**Authors:** Luisa Schmidt, Marc Wiedner, Georgia Velimezi, Jana Prochazkova, Michel Owusu, Sabine Bauer, Joanna I. Loizou

**Affiliations:** a CeMM Research Center for Molecular Medicine of the Austrian Academy of Sciences, Lazarettgasse 14, AKH BT 25.3 1090 Vienna, Austria; b Ludwig Boltzmann Institute for Cancer Research, Waehringer Strasse 13A, 1090 Vienna, Austria

**Keywords:** Replication stress, 53BP1, ATM, ATMIN, MEFs, mouse embryonic fibroblasts, Aph, aphidicolin, IR, ionizing radiation, Gy, Gray, MMS, methyl methanesulfonate, HU, hydroxyurea, NCS, neocarzinostatin, DAPI, diamidino-2-phenylindole, HR, homologous recombination, NHEJ, non-homologous end-joining, CFS, chromosomal fragile sites

## Abstract

Unresolved replication intermediates can block the progression of replication forks and become converted into DNA lesions, hence exacerbating genomic instability. The p53-binding protein 1 (53BP1) forms nuclear bodies at sites of unrepaired DNA lesions to shield these regions against erosion, in a manner dependent on the DNA damage kinase ATM. The molecular mechanism by which ATM is activated upon replicative stress to localize the 53BP1 protection complex is unknown. Here we show that the *ATM*-*IN*teracting protein ATMIN (also known as ASCIZ) is partially required for 53BP1 localization upon replicative stress. Additionally, we demonstrate that ATM activation is impaired in cells lacking ATMIN and we define that ATMIN is required for initiating ATM signaling following replicative stress. Furthermore, loss of ATMIN leads to chromosomal segregation defects. Together these data reveal that chromatin integrity depends on ATMIN upon exposure to replication-induced stress.

## 1 Introduction

Unresolved replication intermediates can occur during S/G2-phases of the cell cycle and can be converted into DNA lesions in M-phase. It has been shown that 53BP1 forms nuclear bodies at such sites of unrepaired DNA lesions in the subsequent G1-phase to shield these regions against erosion [1]. In the following S-phase 53BP1 nuclear bodies are resolved and DNA lesions are repaired. Interestingly, chromosomal fragile sites (CFS) are enriched within regions of the genome that are sensitive to replication-induced stress and as a consequence such sites, including FRA3B and FRA16D, are commonly mutated in cancers [2], [3].

53BP1 nuclear bodies consist of several DNA repair proteins including the ubiquitin ligases RNF8 and RNF168, ATM (autophosphorylated at S1981), NBS (phosphorylated at S343), MDC1 and γH2AX [1]. Moreover, these foci also co-localize with OPT (Oct-1, PTF and transcription) domains that are known to occur in G1 and represent sites of low transcriptional activity, hence functioning to suppress transcription at DNA damage sites [4].

The DNA damage kinase ATM (mutated in the inherited recessive autosomal disease ataxia telangiectasia) [5] has been implicated in 53BP1 localization both in basal conditions and after aphidicolin-induced replicative stress [1], [4]. Aphidicolin is an inhibitor of the replicative polymerase α (and also potentially of polymerase δ) and has been shown to specifically increase the breakage of CFS [4], [6]. Nuclear body formation of 53BP1 in response to aphidicolin-induced replicative stress is thought to suppress the sensitivity of CFS to breakage by shielding these regions against erosion and degradation [1]. Indeed loss of 53BP1 leads to increased breakage within CFS upon replication stress [1].

This requirement for ATM in 53BP1 localization in response to replicative stress has only recently been reported, in contrast to the intensively investigated roles of ATM in response to the generation of DNA double-strand breaks [7]. Canonical ATM signaling following DNA double-strand breaks has been shown to require NBS [8]. In contrast, little is known about ATM activation following other stresses [9]. The *ATM*-*IN*teracting protein (ATMIN; also known as ASCIZ) is required for ATM activation after cellular stresses including chloroquine and hypotonic stress [10]. ATMIN has also been shown to be required for the repair of DNA alkylation damage and as it is required for localization of Rad51 it may link base damage repair with the repair of DNA double-strand breaks by homologous recombination [11], [12].

The molecular mechanism for ATM activation following aphidicolin-induced replicative stress, and the subsequent localization of 53BP1 to replication sensitive sites is not known. In the present study we define a partial requirement for ATMIN in ATM-mediated localization of 53BP1 following replicative stress. Furthermore, we show that ATMIN is required for ATM-dependent signaling and to suppress chromosomal segregation defects and chromosomal instability. These data define ATMIN as a critical mediator in ATM signaling following replicative stress.

## 2 Materials and methods

### 2.1 Cell culture, DNA damage induction and siRNA transfections

Cells were cultured in DMEM (Invitrogen) supplemented with penicillin/streptomycin (Invitrogen), and 10% FCS (Invitrogen). Cells were incubated at 37 °C with 5% CO_2_ and 3% O_2_. Ionizing irradiation (IR) experiments were performed using a Cs137 Gamma Irradiator at the indicated doses followed by a recovery period of 30 min. Aphidicolin, MMS, NCS, HU and topotecan were purchased from Sigma. siRNAs were purchased from Dharmacon and transfected using the Lullaby transfection reagent as directed by the manufacturer (Oz Biosciences). Cells were analyzed 48–72 h after transfection.

### 2.2 Quantitative RT-PCR

RNA was extracted using TRIzol reagent (Life Technologies). RNA was treated with 1 μl DNase (TURBO™ DNase, Lifetechnologies) in a 0.1 volume TURBO DNase Buffer for 30 min at 37 °C. The reaction was terminated with 0.1 volume of DNase inactivation reagent (Life Technologies), incubated for 5 min at room temperature and centrifuged at 10,000 × *g* for 1.5 min before transferring RNA into fresh tubes. RNA was reverse transcribed with the SuperScript III Reverse Transcriptase protocol (Invitrogen) to obtain cDNA. An amount of 50 ng of cDNA template was used with the reverse transcription SYBR Green qPCR Mastermix (QIAGEN). The 7900HT Fast Real-Time PCR System (Applied Biosystems) was used for the quantitative RT-PCR reaction. RNF168: forward primer - GCCTGTGGTGCCGAATG, reverse primer – CCCATGATTGCTTGGTCTTGT. GAPDH: forward primer – CGAGCCACATCGCTCAGACA, reverse primer – GGCGCCCAATACGACCAAAT. The data was normalized to GAPDH.

### 2.3 I-Sce1 assay

Reporter cell lines were used to assess homologous recombination (DR-GFP) and non-homologous end joining (EJ5-GFP) [13]. For transfections, I-SceI expression vector (pCBASce), GFP expression vector (pCAGGS-NZEGFP) and a control empty vector (pCAGGS-BSKX) were used [13]. In detail, 2 × 10^5^ cells were plated into a 12-well dish and transfected with non-targeting or siRNA targeting ATMIN (Dharmacon) using Lullaby reagent (OZ Biosciences). After 48 h, cells were transfected with I-SceI or control plasmids. Transfection complexes were prepared by mixing Lipofectamine 2000 (Life Technologies) in OptiMEM (Invitrogen) with 0.8 μg of expression vectors for I-SceI or control vectors per sample. Samples were analyzed 3 days after transfection by immunoblotting to assess the knockdown efficiency. The frequency of GFP^+^ cells was determined on a Fortessa II flow cytometer (BD Bioscience).

### 2.4 Protein extracts and immunoblotting

Cells were extracted in RIPA lysis buffer (NEB) supplemented with protease inhibitors (Sigma) and phosphatase inhibitors (Sigma, NEB). Immunoblots were performed using standard procedures. Protein samples were separated by SDS–PAGE (3–8% gradient gels; Invitrogen), and subsequently transferred onto nitrocellulose membranes. All primary antibodies were used at 1:1000 dilution, except for P-S957-SMC1 that was used at 1:400, and secondary antibodies at 1:5000. The following antibodies were used: ATM 2C1 (Santa Cruz), P-S1981-ATM (10H11.E12; NEB), ASCIZ (Millipore), P-S824-KAP1 (Bethyl Laboratories, Inc), KAP1 (Bethyl Laboratories, Inc), P-S15-p53 (16G8; NEB), P-S957-SMC1 (5D11G5; Millipore), SMC1 (Abcam), P-S317-CHK1 (NEB), CHK1 (DCS-310; Santa Cruz), FANCD2 (EPR2302; Abcam), p95/NBS (NEB), β-actin (Sigma), 53BP1 (H300; Santa Cruz), Chk1 (DCS-310; Santa Cruz), HRP-conjugated goat anti-mouse/rabbit IgG (Sigma).

### 2.5 Immunofluorescence, microscopy and statistics

Cells were adhered onto coverslips and stained as described previously [20]. The antibodies used were 53BP1 (H300; Santa Cruz) and Alexa Fluor^**®**^ 546 goat anti-rabbit or Alexa Fluor^**®**^ 488 goat anti-rabbit (Invitrogen). Fixed cells were counterstained with diamidino-2-phenylindole (DAPI). Images of cells were acquired on a Deconvolution Microscope (Leica). Cell Profiler cell image analysis software (developed by the Broad Institute) was used for the quantification of 53BP1 focus formation. Intensity of 53BP1 was measured using the Thermo Scientific Cellomics high content screening platform where the intensity of individual foci per nucleus was assessed. Statistical significance was calculated using Fisher's exact test.

### 2.6 Cell proliferation

ATMIN^+/+^ and ATMIN^Δ/Δ^ MEFs were seeded at a density of 2 × 10^5^ per well in a 24-well plate. Cells were collected after 48 h, counted and 2 × 10^5^ of cells were replated. Cells were counted at 3 consecutive passages.

### 2.7 Cell cycle analysis

After treatments as indicated, cells were fixed with 70% ethanol, rehydrated in PBS, stained with propidium iodide and analyzed on a FACScalibur flow cytometer. Following cell acquisition, analysis was performed using FlowJo software (Tree Star).

### 2.8 Cell survival analysis

Cells were seeded at a density of 7 × 10^3^ cells per well in a 96-well-plate. On the following day, cells were treated as indicated and consequently grown in drug-free media until control cells reached 90% confluence. Aphidicolin was used for 24 h, MMS was used for 1 h and HU was used for 24 h. Cells were washed twice with PBS upon which 50 μl of CellTiter-Glo^**®**^ Reagent (Promega) was added. Following 30 min of gentle agitation, luminescence was recorded using a Victor™ X3 Multilabel Plate Reader (PerkinElmer). Data was analyzed using GraphPad Prism^**®**^ software.

### 2.9 Colony formation assay

Cells were seeded at a density of 250 cells per 6-well and treated 24 h later with aphidicolin, HU, NCS, topotecan or MMS at the indicated concentrations. Aphidicolin, NCS and topotecan were left on for 5 days. HU treatment was for 24 h and MMS treatment was for 1 h after which cells were fixed 7 days later. Cells were then washed with PBS, fixed with 3.7% Formaldehyde solution and stained with 0.1% Crystal Violet (Sigma–Aldrich, diluted in ethanol). After scanning the plates, the Crystal Violet was extracted from cells with 50% ethanol and the absorbance was measured at 595 nm on a spectrophotometer.

## 3 Results

### 3.1 ATMIN is partially required for 53BP1 localization after replicative stress

**Fig. 1:**
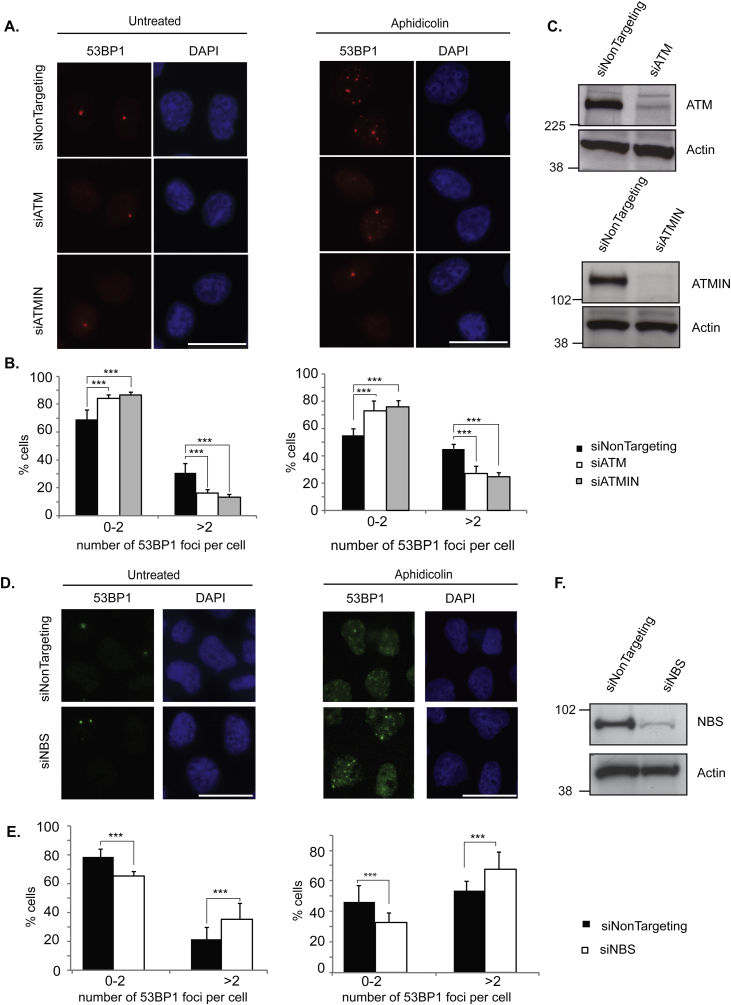
Depletion of ATMIN but not NBS partially impairs 53BP1 localization to sites of replication-induced stress. (A) HeLa cells were depleted for ATM (siATM) or ATMIN (siATMIN), or treated with non-targeting siRNA (siNonTargeting). Cells were then either left untreated or treated with aphidicolin (1 μM for 24 h) and subsequently stained for 53BP1. DNA was visualized by DAPI staining. (B) Quantification of 53BP1 foci per cell in (A). (C) The siRNA-mediated depletion of ATM and ATMIN was confirmed by immunoblotting. (D) HeLa cells were depleted for NBS, either left untreated or treated with aphidicolin as in (A) and stained for 53BP1. DNA was visualized by DAPI staining. (E) Quantification of 53BP1 foci per cell in (D). (F) The siRNA-mediated depletion of NBS was confirmed by immunoblotting. Scale bars denote 20 μm. siNonTargeting was used as a negative control. ***p < 0.001. Between 200 and 1000 cells were analyzed per condition. Statistical significance was calculated using Fisher's exact test.

To determine the molecular mechanism by which ATM functions in 53BP1 nuclear body formation we assessed the role of the ATM co-factors ATMIN and NBS in this process. First, we depleted either ATM (as a positive control) or ATMIN in HeLa cells by siRNA and assessed the ability of 53BP1 to localize to sites of replication-induced stress. Similar to ATM, depletion of ATMIN resulted in a partially diminished ability of 53BP1 to form nuclear bodies both in basal conditions and after induction of replicative stress by aphidicolin (Fig. 1A and B). The decreased ability of 53BP1 to localize to damage sites was equally impaired upon loss of either ATM or its co-factor ATMIN. The knockdown of both ATM and ATMIN was confirmed by immunoblotting (Fig. 1C). We also assessed the mean intensity of 53BP1 foci following aphidicolin treatment in cells depleted for ATMIN (siATMIN) (Supplementary Fig. 1A) in comparison to cells depleted for 53BP1 or RNF168, as negative controls. RNF168 is a ubiquitin ligase that is recruited to sites of DNA damage by binding to ubiquitinated histone H2A and H2AX and amplifies the RNF8-dependent H2A ubiquitination, promoting the formation of ‘Lys-63’-linked ubiquitin conjugates [21]. This in turn leads to 53BP1 recruitment [21]. As non-targeting siRNA controls we used either cells not treated with siRNA (no siRNA), a non-targeting RNA (siNonTargeting) or an siRNA that cannot be processed by the RISC complex (siRiscFree). We observed that depletion of ATMIN resulted in a significant decrease in 53BP1 intensity. The depletion of 53BP1 was confirmed by immunoblotting (Supplementary Fig. 1B) and the depletion of RNF168 was confirmed by quantitative RT-PCR (Supplementary Fig. 1C).

We next assessed the requirement of NBS in ATM-mediated 53BP1 recruitment to sites of replication stress-induced damage. We observed that reduction of NBS led to an increased number of 53BP1 nuclear bodies in nuclei of untreated cells and after treatment of cells with aphidicolin (Fig. 1D and E). The knockdown of NBS was confirmed by immunoblotting (Fig. 1F). These data indicate that ATMIN, and not NBS, partially diminishes ATM-dependent 53BP1 localization at sites of replicative stress.

Since pools of siRNAs were used in Fig. 1 and Supplementary Fig. 1, we next confirmed these findings with individual independent siRNAs for ATMIN, ATM and NBS. We identified at least two siRNAs that resulted in depletion of ATMIN, NBS or ATM (Supplementary Fig. 2A) and proceeded to determine the effects of depleting these proteins on 53BP1 focus formation following aphidicolin treatment (Supplementary Fig. 2B). Indeed, we confirmed that whereas depletion of ATMIN or ATM resulted in a decreased ability to localize 53BP1, depletion of NBS resulted in elevated localization of 53BP1.

**Fig. 2:**
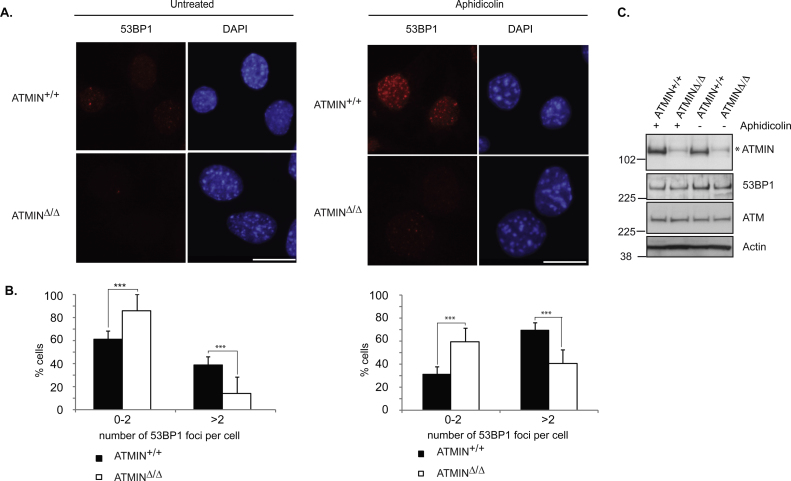
ATMIN-deficient murine cells display a diminished capacity in relocalising 53BP1 upon aphidicolin treatment. (A) ATMIN^+/+^ and ATMIN^Δ/Δ^ MEFs were either left untreated or treated with aphidicolin (1 μM for 24 h) and subsequently stained for 53BP1. DNA was visualized by DAPI staining. (B) Quantification of 53BP1 foci per cell in (A). (C) Untreated or aphidicolin treated (1 μM for 24 h) ATMIN^+/+^ and ATMIN^Δ/Δ^ MEFs were analyzed by immunoblotting with the indicated antibodies. *A non-specific band. Scale bar in A is 20 μm. ***p < 0.001. Between 300 and 700 cells were analyzed per condition. Statistical significance was calculated using Fisher's exact test.

To confirm that ATMIN is required for 53BP1 localization we turned to murine cells. We treated ATMIN proficient (ATMIN^+/+^) and deficient (ATMIN^Δ/Δ^) mouse embryonic fibroblasts (MEFs) with or without aphidicolin and assessed 53BP1 localization by immunofluorescence. As expected, we observed a reduction in the number of nuclei with more than two 53BP1 foci, both in basal conditions and after aphidicolin treatment, upon loss of ATMIN (Fig. 2A and B). We did not observe any effects at the protein level of either ATM or 53BP1 upon loss of ATMIN, indicating that ATMIN ablates the ability of 53BP1 to localize to nuclear bodies without affecting the protein levels of either 53BP1 or the upstream kinase ATM (Fig. 2C).

### 3.2 ATM-kinase activity in response to aphidicolin is ATMIN dependent

**Fig. 3:**
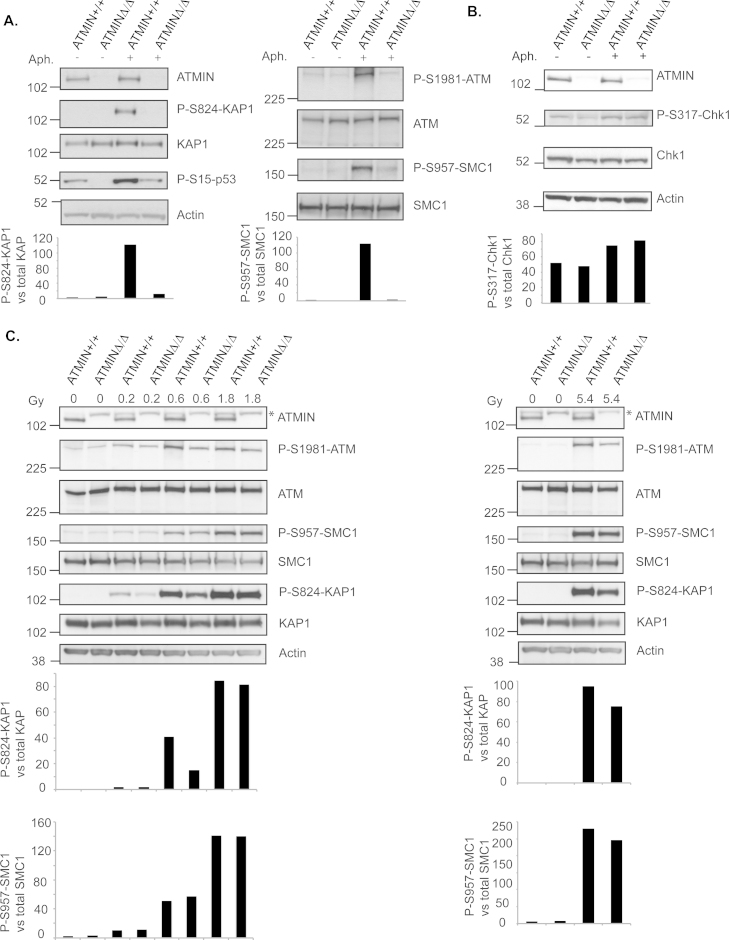
ATMIN is required for ATM-mediated signaling of replicative stress. (A, B) ATMIN^+/+^ and ATMIN^Δ/Δ^ MEFs were either left untreated or treated with 1 μM aphidicolin (Aph.) overnight, followed by analysis by immunoblotting as indicated. (C) ATMIN^+/+^ and ATMIN^Δ/Δ^ MEFs were either left untreated or exposed to the indicated doses (0.2, 0.6, 1.8 and 5.4 Gy) of ionizing radiation, in Gray (Gy), and incubated for 30 min, followed by analysis via immunoblotting. Quantification of immunoblotting displayed in (A–D) was analyzed with the ImageJ software. Values represent relative band area of (A). P-S824-KAP1 protein levels normalized to total KAP1 protein levels (arbitrary units) as well as P-S957-SMC1 protein levels normalized to total SMC1 protein levels (arbitrary units); (B) P-S317-Chk1 protein levels normalized to total Chk1 protein levels (arbitrary units); (C) P-S824-KAP1 protein levels normalized to total KAP1 protein levels (arbitrary units) as well as P-S957-SMC1 protein levels normalized to total SMC1 protein levels (arbitrary units). *A non-specific band.

To determine whether loss of ATMIN not only reduces 53BP1 localization but also ATM-mediated signaling after aphidicolin, we treated ATMIN^+/+^ or ATMIN^Δ/Δ^ MEFs, with or without aphidicolin and assessed ATM-kinase activation by its autophosphorylation (S1981) and the phosphorylation of its targets KAP1 (S824), p53 (S15) and SMC1 (S957) (Fig. 3A). We observed a dramatic decrease in the phosphorylation of S824-KAP1, S15-p53, S957-SMC1 and S1981-ATM upon loss of ATMIN following aphidicolin treatment, while the total levels of these proteins remained unchanged. The requirement for ATMIN in the activation of ATM appeared to be specific since under the same conditions the phosphorylation of the ATR target Chk1 (at S317) was not impaired upon loss of ATMIN (Fig. 3B). These data suggest that ATMIN is required for ATM (and not ATR) activation after aphidicolin-induced replicative stress.

**Fig. 4:**
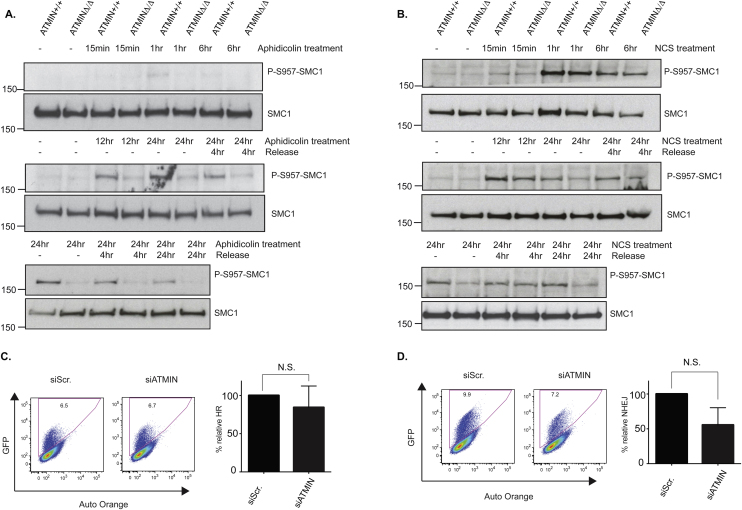
ATMIN is required for initiating ATM-mediated signaling following replication stress. (A) ATMIN^+/+^ and ATMIN^Δ/Δ^ MEFs were either left untreated or treated with 1 μM aphidicolin for 15 min, 1 h, 6 h, 12 h or 24 h. After the 24-h treatment, aphidicolin was removed from the cells and cells were incubated in drug-free media for 4 h or 24 h. Resulting cell extracts were probed for P-S957-SMC1 or total SMC1, as indicated. B ATMIN^+/+^ and ATMIN^Δ/Δ^ MEFs were treated as in (A) using neocarzinostatin (NCS) at 50 ng/ml instead of aphidicolin. (C, D) DR-GFP U2OS cells (C) and EJ5-GFP U2OS cells (D) were transfected with siATMIN or siScr. (siScrambled). After 48 h, cells were transfected with the I-SceI-expressing vector. Cells were harvested 3 days after I-SceI transfection and the expression of GFP-positive cells was assessed by flow cytometry. Data quantification was performed from 3 independent experiments using Student's *t* test.

Measurement of ATM-kinase activation by phosphorylation of its substrates, following the induction of ionizing radiation (IR)-induced DNA double-strand breaks was found to be affected to a lesser degree in the absence of ATMIN (Fig. 3C). We next investigated this finding further and determined whether ATMIN is required for the initiation or persistence of ATM-mediated signaling following induction of replicative stress and DNA double-strand breaks. Hence, we firstly treated ATMIN^+/+^ or ATMIN^Δ/Δ^ MEFs with aphidicolin for increasing time points, up to 24 h. We observed that initiation of SMC1 phosphorylation was impaired in ATMIN-deficient MEFs. This decrease in ATM signaling was maintained during a recovery period of up to 24 h (Fig. 4A and Supplementary Fig. 3A).

As we had observed a reduction in the phosphorylation of KAP1 following treatment of cells with ionizing radiation (at 0.6 Gy and 5.4 Gy), we next determined if persistence or initiation of signaling was affected in response to DNA double-strand breaks. This was additionally important as in Fig. 3 the aphidicolin treatment was for 24 h whereas the IR recovery was for 30 min. For this purpose we treated ATMIN^+/+^ or ATMIN^Δ/Δ^ MEFs with the radiomimetic neocarzinostatin (NCS) at 50 ng/ml under the same conditions as for aphidicolin (Fig. 4B and Supplementary Fig. 3B). We observed that initiation of ATM signaling was slightly more pronounced in the ATMIN-deficient MEFs (at 1 h and 6 h post-NCS treatment). However at later time points (12 h and 24 h post-aphidicolin treatment) and in the recovery periods (4 h and 24 h) the phosphorylation of SMC1 was reduced in ATMIN^Δ/Δ^ MEFs in comparison to wild-type cells (Fig. 4B and Supplementary Fig. 3B). This data reveal that ATMIN-deficient cells can activate ATM leading to SMC1 phosphorylation with heightened kinetics in comparison to wild-type MEFs. Furthermore, the clearance of signaling DNA double-strand breaks appears to be faster in ATMIN-deficient cells in comparison to wild-type MEFs.

We went on to determine whether loss of ATMIN does affect DNA double-strand break repair by assessing the repair of an I-Sce1 induced DNA lesion. In this regard, we assessed the ability of U2OS cells depleted for ATMIN to repair an I-Sce1 induced DNA double-strand break by either homologous recombination (HR) or non-homologous end joining (NHEJ) [13]. Although we did not observe a statistically significant effect on either HR (Fig. 4C) or NHEJ (Fig. 4D) upon depletion of ATMIN using this approach, we did consistently observe reduced NHEJ in cells depleted for ATMIN.

We next assessed sensitivity of ATMIN-deficient MEFs to a broad range of DNA damaging agents either by measuring ATP levels, which signal the presence of metabolically active cells (Supplementary Fig. 4), or by colony formation assays (Supplementary Figs. 5–6). In the former assay we did not observe a sensitivity of ATMIN-deficient cells to either the alkylating agent *methyl* methanesulfonate (MMS) or hydroxyurea (HU), that induces replication stress by depleting the pool of deoxynucleotides via inhibition of ribonucleotide reductase **(**Supplementary Fig. 4). As this is a relatively short-term assay of three days, we next assessed cellular sensitivity via colony formation (for 5–7 days, as indicated), in response to DNA damaging agents. To this end we treated wild-type MEFs or MEFs lacking either ATM or ATMIN with the replication-inducing stress agents aphidicolin and HU, the radiomimetic agent NCS, the alkylating agent MMS and the topoisomerase 1 inhibitor topotecan (Supplementary Figs. 5–6). Although some sensitivity of ATMIN-deficient MEFs was observed following treatment with aphidicolin, this was not the case for HU treatment. We did not observe sensitivity to NCS (Supplementary Fig. 5). However, we did observe sensitivity to topotecan. Finally, we confirmed the already published sensitivity of ATMIN-deficient MEFs to MMS via this longer-term assay [11] (Supplementary Fig. 6).

### 3.3 ATM-independent role of ATMIN in clearing replication stress

**Fig. 5:**
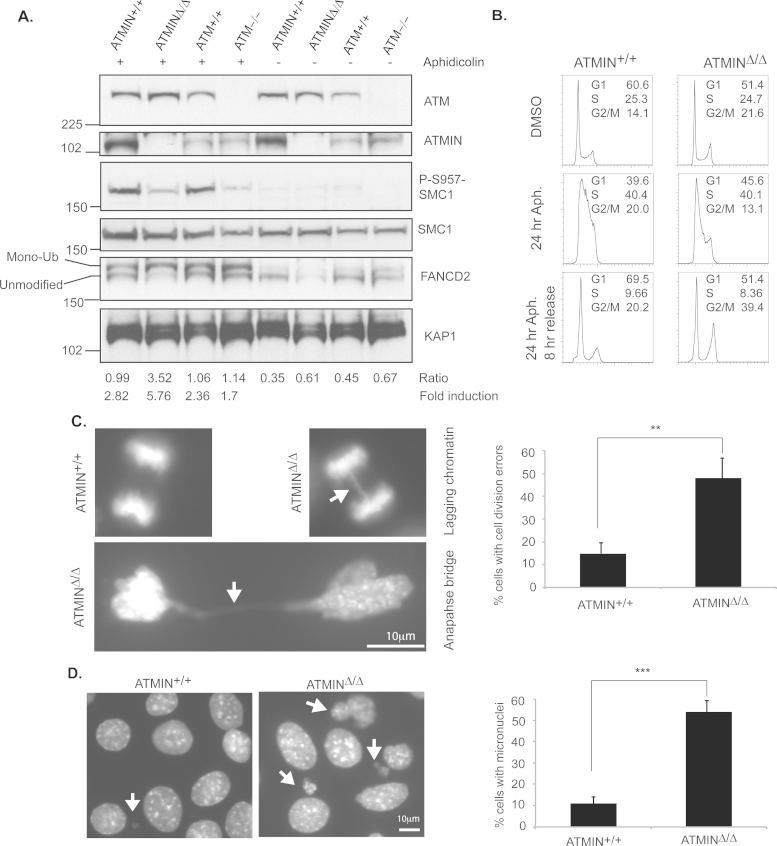
Proper cell cycle progression and chromosomal segregation depends on ATMIN following aphidicolin treatment. (A) ATMIN^+/+^ and ATMIN^Δ/Δ^ MEFs were treated with 1 μM aphidicolin for 24 h followed by analysis by immunoblotting with the indicated antibodies. For FANCD2 ‘Ratio’ denotes the amount of monoubiquitinated (Mono-Ub) compared to unmodified FANCD2. ‘Fold induction’ denotes the increase in monoubiquitinated FANCD2 in aphidicolin treated samples as compared with the corresponding control treated sample. (B) ATMIN^+/+^ and ATMIN^Δ/Δ^ MEFs were either treated with DMSO, 1 μM aphidicolin (Aph.) for 24 h or 1 μM aphidicolin (Aph.) for 24 h followed by incubation in drug-free media for 8 h (8 h release) and cell cycle profiles were analyzed by propidium iodide staining. C. ATMIN^+/+^ and ATMIN^Δ/Δ^ MEFs were treated with 1 μM aphidicolin for 24 h followed by incubation in drug-free media for 8 h and stained with DAPI. Defects in cell division marked by lagging chromosomes and anaphase bridges were imaged and quantified as were cells displaying formation of micronuclei (D). At least 200 cells were analyzed per condition. **p < 0.01, ***p < 0.001.

We next wanted to define the cellular effect of diminished 53BP1 focus formation and ATM activation (as indicated by phosphorylation of KAP1, p53, SMC1 as well as ATM-autophosphorylation) following aphidicolin treatment. Hence, wild-type, ATMIN-deficient or ATM-deficient MEFs were left untreated or treated with aphidicolin and the amount of monoubiquitinated FANCD2 was determined by calculating the fold induction of monoubiquitinated compared to unmodified FANCD2 (Fig. 5A). The S-phase checkpoint facilitates damage-induced monoubiquitination of FANCD2 and recruitment to sites of replication stress [22]. Hence, this finding indicates that cells lacking ATMIN have increased aphidicolin-induced replication stress in comparison to control MEFs. Therefore, these data show that ATMIN is important in the resolution of replication stress. However, it was apparent that ATM-deficient MEFs have relatively normal levels of monoubiquitinated FANCD2. These data indicate that ATMIN has functions independent of ATM in clearing replication stress.

### 3.4 ATMIN is required for cell cycle progression and chromosome segregation following replication stress

We next investigated the cellular consequences of the loss of ATMIN upon aphidicolin-induced replicative stress. Hence, we treated wild-type MEFs or MEFs lacking ATMIN (ATMIN^Δ/Δ^), with or without aphidicolin for 24-h and measured cell cycle progression following release into compound-free media (Fig. 5A). Loss of ATMIN impaired cell cycle progression recovery following aphidicolin-induced stress. We observed that at 8-h post-aphidicolin release wild-type MEFs had returned to a normal cell cycle profile. However, MEFs lacking ATMIN failed to resume normal cell cycle progression within this time frame. A normal cycle progression was observed in ATMIN^Δ/Δ^ cells at 24-h post-release into drug-free media (Supplementary Fig. 7A). Therefore, these data indicate that ATMIN is required for the resumption of normal cell cycle kinetics following replication stress. However, loss of ATMIN does not affect cell proliferation in basal conditions (Supplementary Fig. 7B).

As a consequence of the inability of ATMIN-deficient cells to clear replication-induced stress, these cells displayed an increase in lagging chromatin and anaphase bridges after aphidicolin treatment (Fig. 5C). As a result of these segregation defects ATMIN-deficient cells showed enhanced in micronuclei formation, an indication of chromosomal instability (Fig. 5D). The segregation defects displayed in ATMIN-deficient cells may be due to many deficiencies including reduced KAP1 and SMC1 phosphorylation as well as reduced 53BP1 localization.

## 4 Discussion

Here, we uncover a novel requirement for ATMIN in partially localizing 53BP1 to sites of replication-induced stress (Fig. 1, Fig. 2 and Supplementary Figs. 1 and 2). Furthermore, we show that this recruitment occurs independently of NBS (Fig. 1 and Supplementary Fig. 2). An absence of a role for NBS in the recruitment of 53BP1 to sites of replication-induced stress is in line with a study from the Nussenzweig group where it was stated that murine B cells lacking NBS display an elevation in 53BP1 focus formation [8]. This is consistent with the findings we present here, indicating that reduction of NBS results in increased 53BP1 nuclear bodies, both in basal conditions and after aphidicolin treatment.

The increase in 53BP1 nuclear bodies upon reduction of NBS could be explained by competition between NBS and ATMIN for ATM binding in order to regulate ATM function [24]. Thus, the absence of NBS increases flux through ATMIN-mediated ATM activation following replicative stress leading to increased 53BP1 nuclear bodies. This competition model of ATMIN and NBS for ATM binding also explains the specificity of ATMIN for ATM activation following replicative stress and for NBS following the induction of DNA double-strand breaks.

Our findings further reveal that ATMIN is required for ATM activity as indicated by an inability of ATM to phosphorylate its targets following replication stress induction in the absence of ATMIN (Fig. 3A). We show that ATMIN is required in the initiation of ATM-signaling following aphidicolin treatment (Fig. 4A). Strikingly, ATMIN appears to be dispensable for ATR-mediated activation under the same stress conditions (Fig. 3B). These data support the hypothesis that ATMIN is specifically required for ATM (and not ATR) function only after aphidicolin-induced stress.

As we did observe a faster clearance of phosphorylated SMC1 in MEFs lacking ATMIN at longer time points following the induction of DNA double-strand breaks via the use of the radiomimetic NCS (Fig. 4B), we asked whether this could lead to a biologically important outcome. For this purpose we assessed the ability of cells with depleted ATMIN to repair a DNA double-strand break induced by the restriction endonuclease I-Sce1 by either HR or NHEJ. Although we did not observe a significant decrease in ATMIN-depleted cells to repair such lesions using this assay we did observe a consistent reduction in NHEJ in cells depleted for ATMIIN (Fig. 4C and D). We cannot exclude that this reduction might lead to a biological effect. It should be noted however that we did not observe a sensitivity of ATMIN-null MEFs to the DNA double-strand break-inducing agent NCS (Supplementary Fig. 5C).

Importantly, since ATMIN has been reported to function as a transcription factor [23], [25], [26] we assessed total protein levels of the ATM/ATR targets that we investigated at the level of phosphorylation as a readout of ATM/ATR activity. We did not find a reduction in any of the tested proteins, indicating that loss of ATMIN does not affect the transcription of these proteins (Fig. 2, Fig. 3).

A recent study from the Heierhorst lab also confirms that ATMIN plays no role in the activation of ATM after the generation of DNA double-strand breaks by IR or after HU-induced stress [25]. We did not assess the requirement for ATMIN in either ATM-mediated signaling or 53BP1 focus formation after HU treatment. However, we observed no sensitivity of ATMIN-deficient cells to HU, either by measuring ATP levels as a readout of cell metabolism or by colony formation (Supplementary Figs. 4B and 5B). We did observe a mild sensitivity to aphidicolin (Supplementary Fig. 5A). The finding that ATMIN is required for cell survival and signaling after aphidicolin treatment but not after HU treatment is intriguing. An explanation could be that aphidicolin and HU treatments do not lead to the same cellular effects: whereas aphidicolin induces CFS, HU is less specific at doing so [27]. Furthermore, HU does not significantly affect the incidence of 53BP1 nuclear bodies whereas aphidicolin does [4]. These differences could indicate that ATMIN is specific for the effects induced by aphidicolin, i.e. 53BP1 nuclear body formation at CFS, and not for the effects induced by HU.

It has also been reported that ATMIN is required for RAD51 focus formation after DNA alkylation damage, induced by *methyl* methanesulfonate (MMS), further supporting the finding that ATMIN plays a role in the DNA damage response [11]. We could reproduce the sensitivity of ATMIN-null MEFs to MMS using colony formation as a readout but not by measuring ATP levels as a readout of cell metabolism (Supplementary Figs. 4A and 6B). This discrepancy could be explained by the fact that the former assay is a shorter term assay of 3 days whereas the latter is a longer-term assay of 7 days. We also assessed sensitivity to topotecan, a topoisomerase 1 inhibitor, and observed a sensitivity of ATMIN-null MEFs to this agent (Supplementary Fig. 6A and B).

ATMIN may contribute to the clearance of replication stress in a manner independent of ATM. This is especially interesting considering that ATM-deficient MEFs have similar levels of ubiquitylated FANCD2 compared to wild-type MEFs. Conversely, ATMIN-deficient MEFs have increased levels of ubiquitylated FANCD2 following aphidicolin treatment which is indicative of enhanced replicative stress (Fig. 5A).

We have recently shown that ATMIN functions as a tumor suppressor in B cells [28]. It is tempting to hypothesize that the mechanism by which ATMIN functions to maintain genomic stability in B cells is via its ability to recruit 53BP1 to CFS. The outcome of defective 53BP1 nuclear body formation in the absence of ATMIN would be decreased shielding of unresolved replication intermediates, structures that commonly occur at CFS. As a consequence, these regions of the genome would become particularly prone to breakage, an event frequently seen in malignancies [2]. Our finding, that loss of ATMIN leads to increased segregation errors and micronuclei formation upon replicative stress (Fig. 5C and D), supports this hypothesis. However, whether ATMIN-deficient tumors carry increased DNA breaks within CFS remains to be seen. Furthermore, whether ATMIN itself is localized to CFSs is still an open question.

## Author contributions

LS, MW, GV, MO, SB and JIL performed the experiments and analyzed the data based on immunoblotting and fluorescence microscopy. MO generated and analyzed Q-RT-PCR data. MW, MO and JIL performed the experiments and analyzed the data for cell survival. JP performed and analyzed the FACS and cell proliferation experiments. JP performed and analyzed the I-Sce1 assays. JIL performed and analyzed cell division errors and micronuclei formation. JIL conceived and supervised the study and wrote the manuscript.

## Conflict of interest

The authors declare that they have no conflict of interest.
