# Supplementary material for: ATMIN is required for the ATM-mediated signaling and recruitment of 53BP1 to DNA damage sites upon replication stress
Source: DNA Repair (Amst). 2014 Dec;24:122–30. doi: 10.1016/j.dnarep.2014.09.001 (PMC4251980; doi:10.1016/j.dnarep.2014.09.001)
Supplement: Supplementary file 1 [file mmc1.pdf]

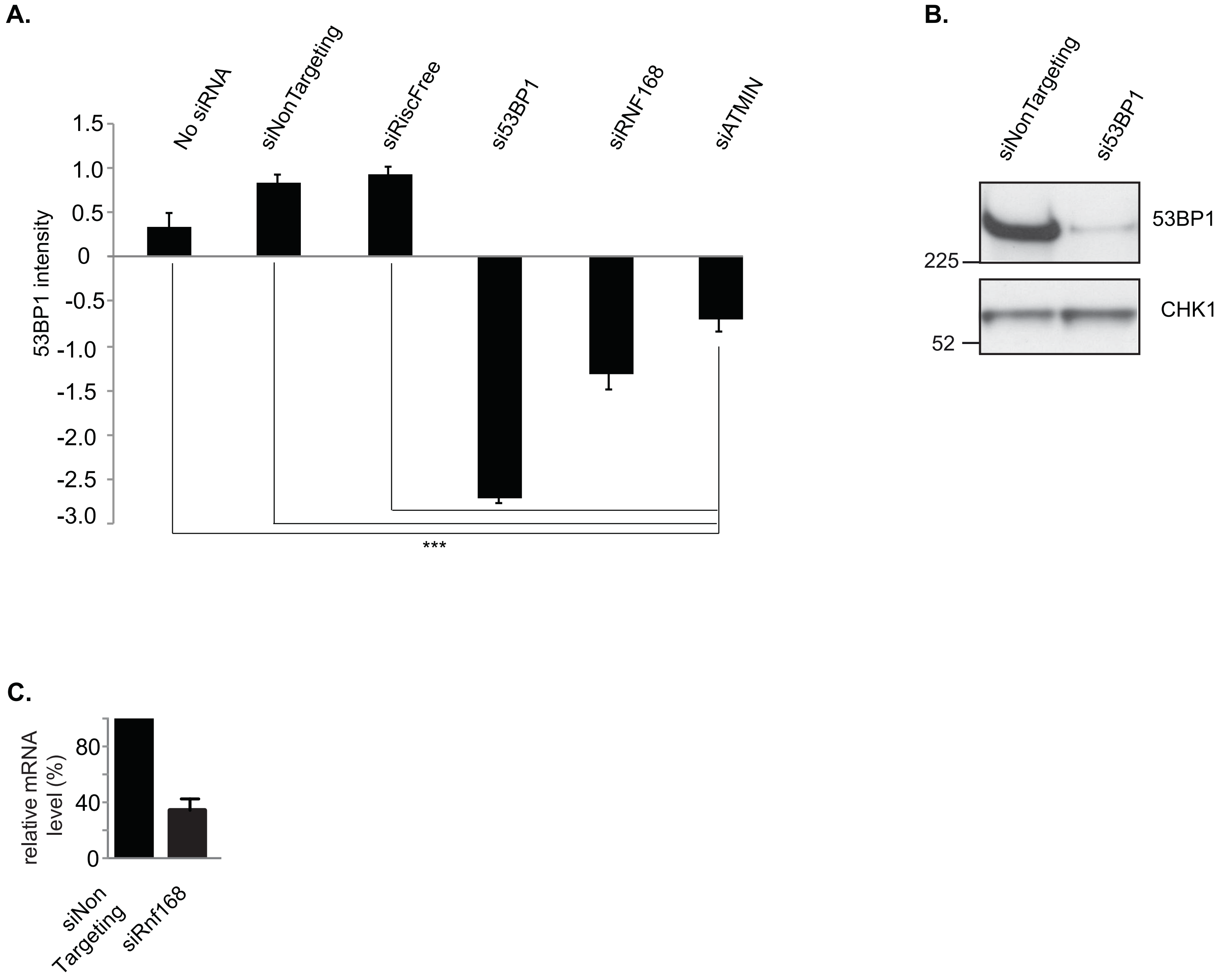

Supplementary Figure 1

A.

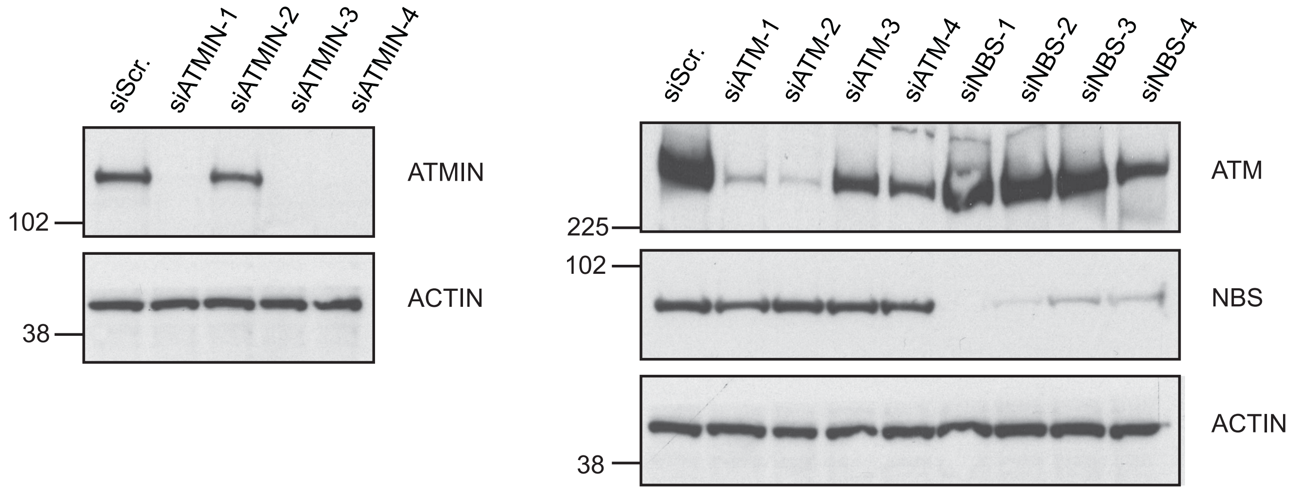

B.

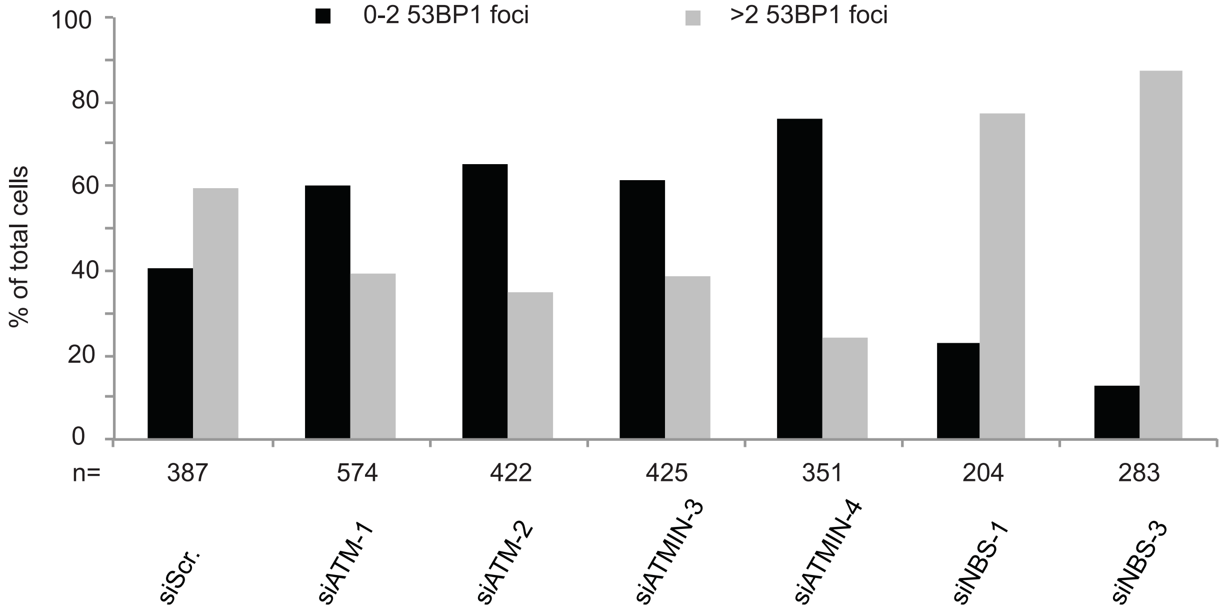

Supplementary Figure 2

A.

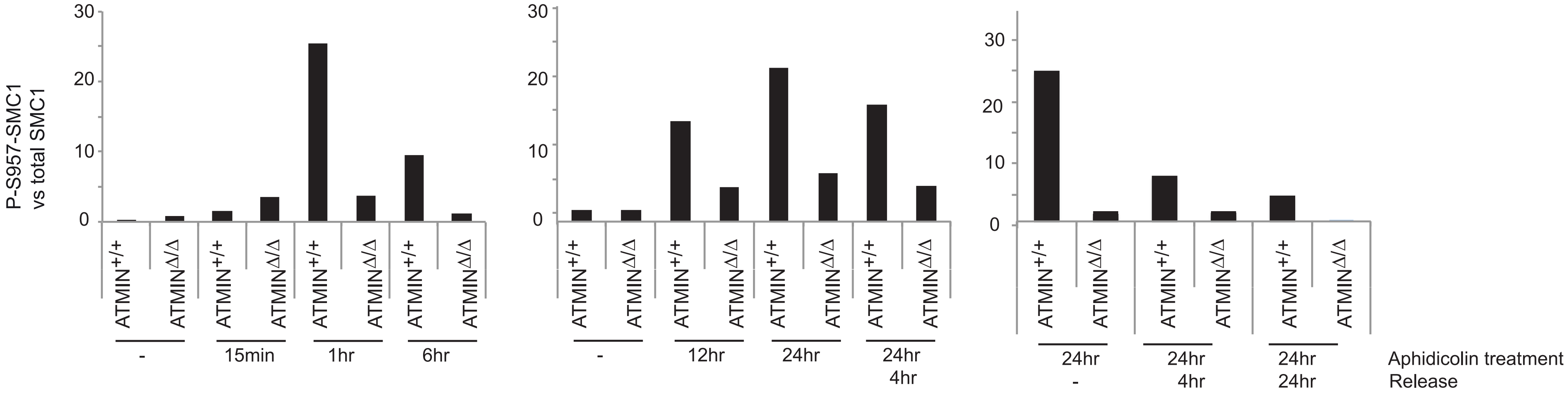

B.

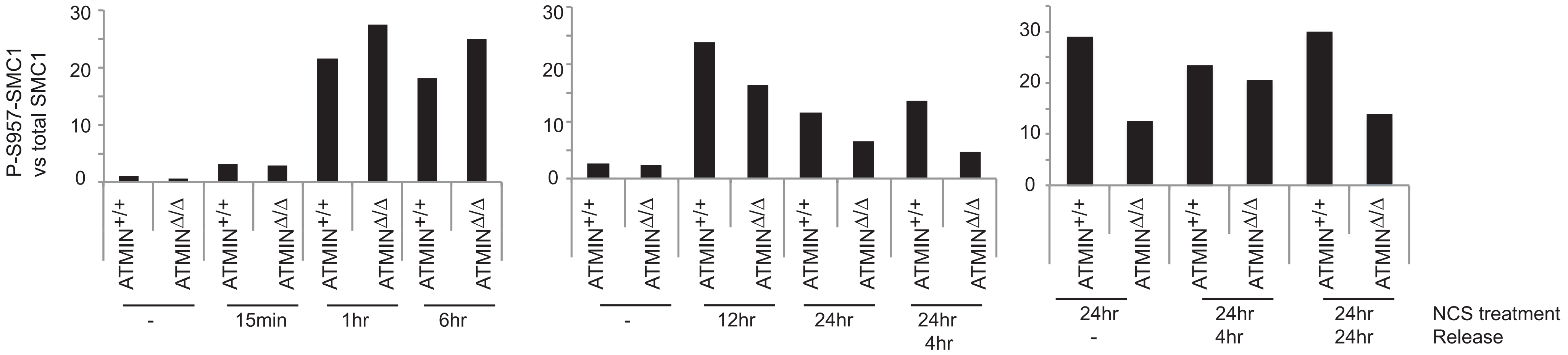

Supplementary Figure 3

A.

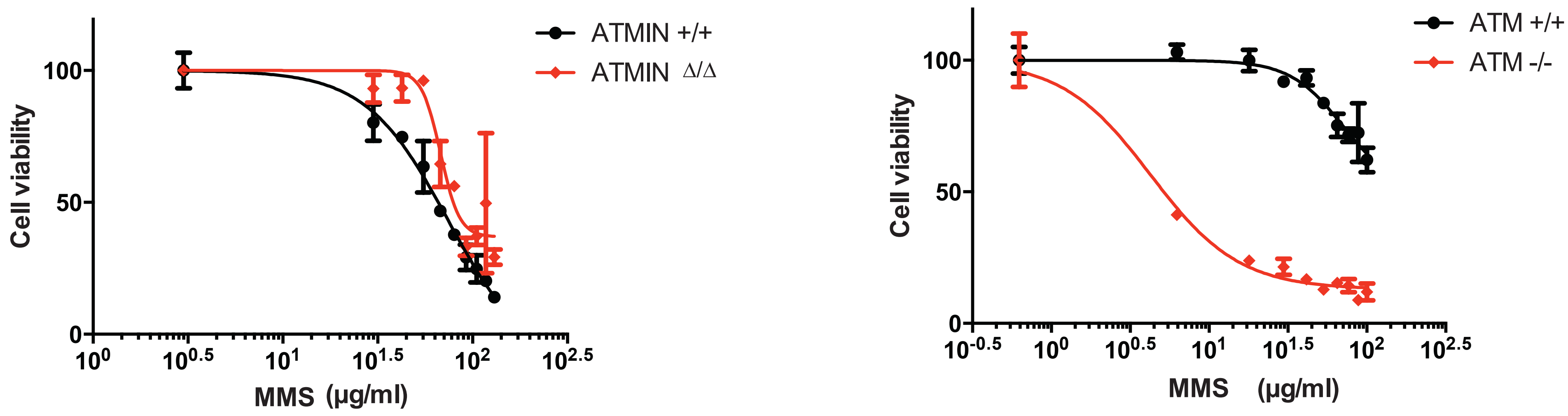

B.

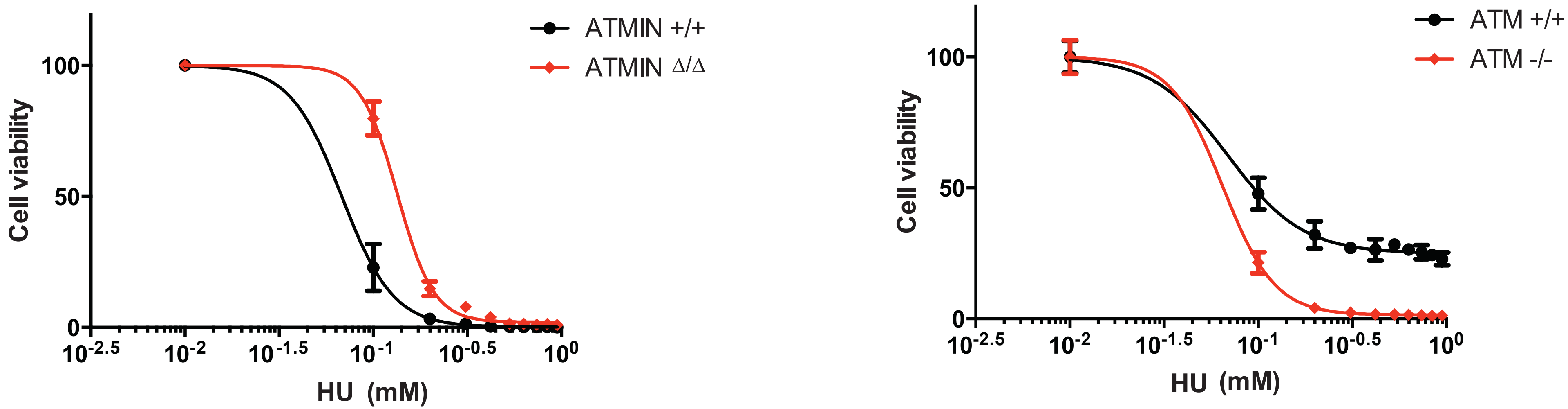

Supplementary Figure 4

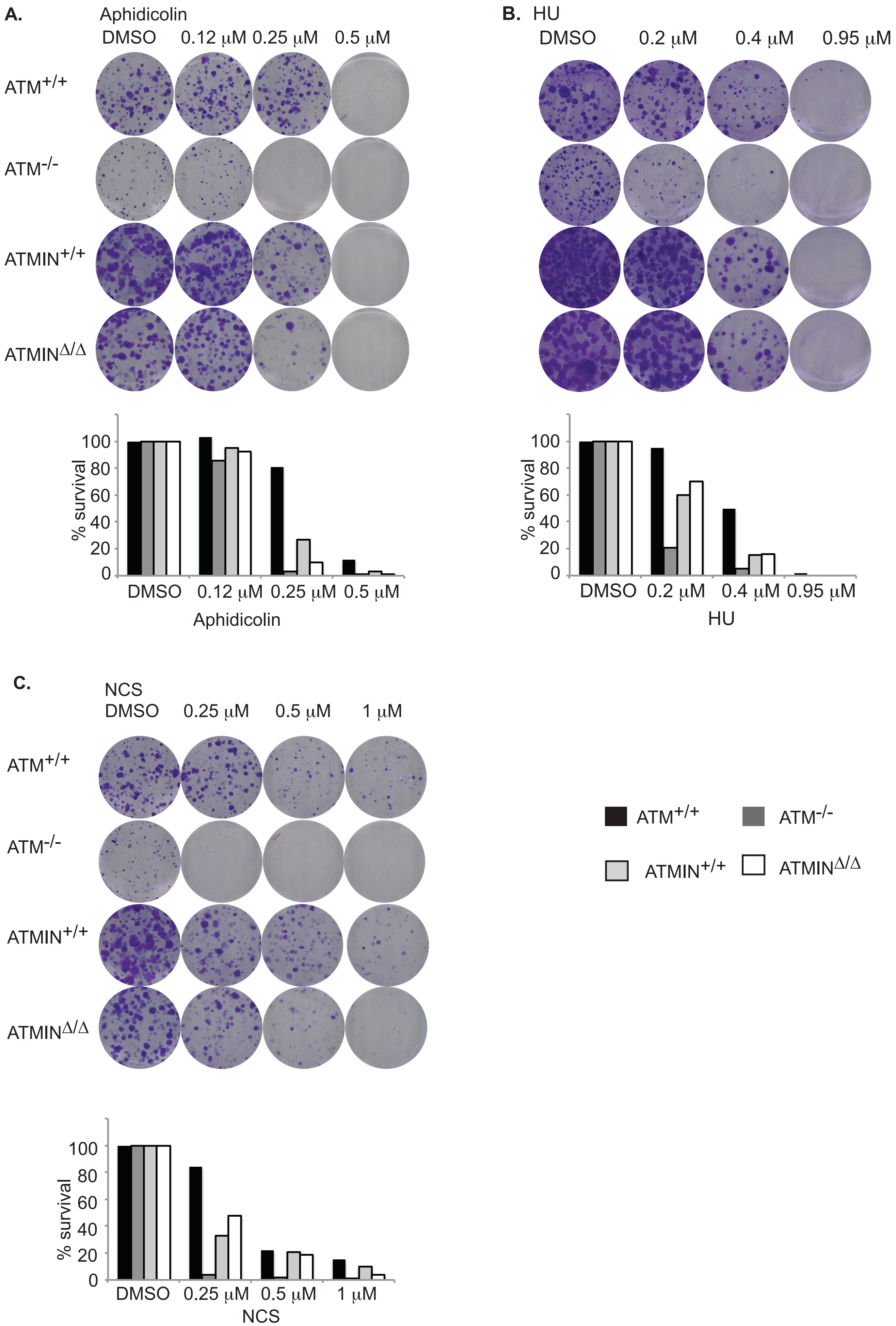

Supplementary Figure 5

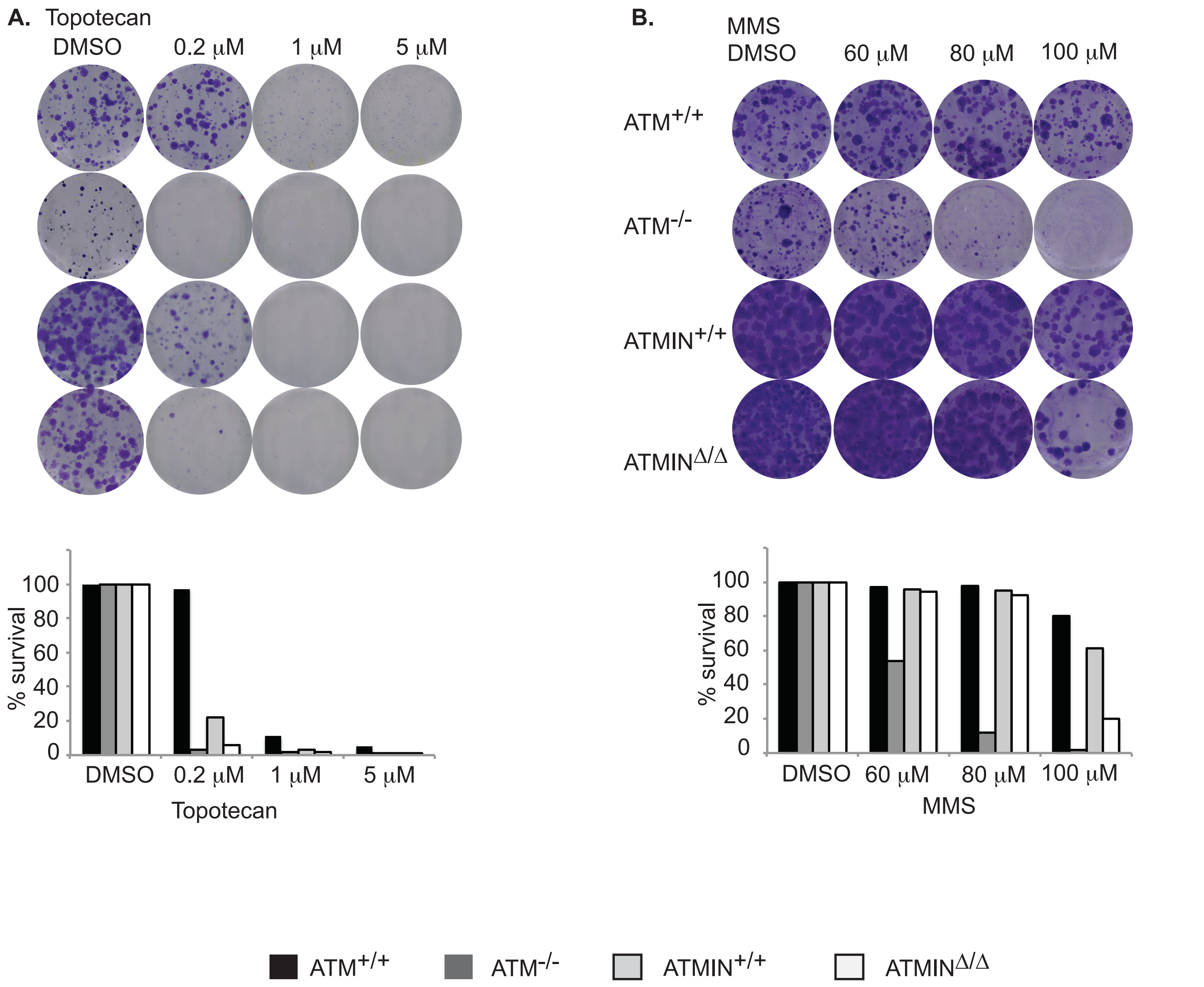

Supplementary Figure 6

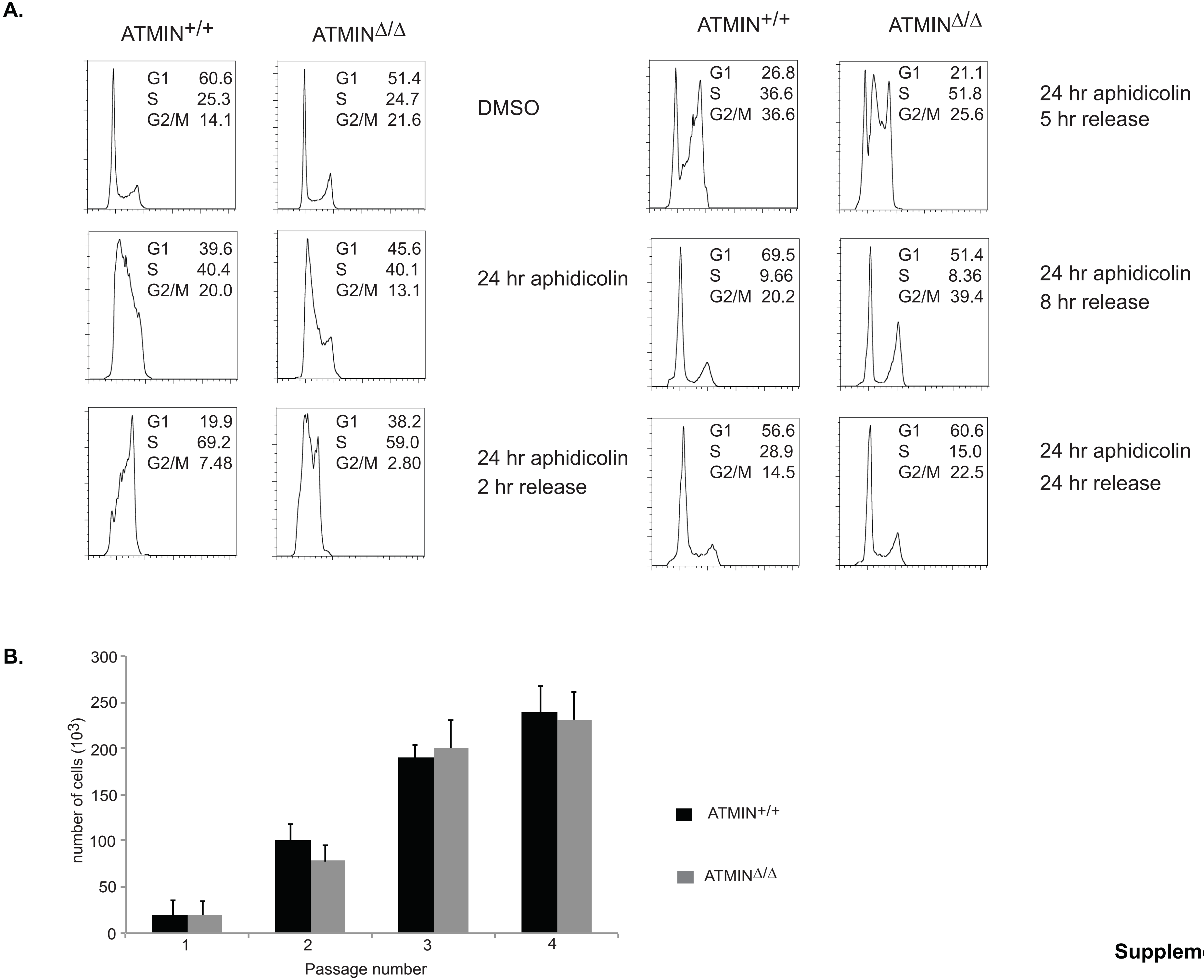

Supplementary Figure 7
